# GABA enhancement by simple carbohydrates in yoghurt fermented using novel, self-cloned *Lactobacillus plantarum* Taj-Apis362 and metabolomics profiling

**DOI:** 10.1038/s41598-021-88436-9

**Published:** 2021-05-03

**Authors:** Farah Salina Hussin, Shyan Yea Chay, Anis Shobirin Meor Hussin, Wan Zunairah Wan Ibadullah, Belal J. Muhialdin, Mohd Syahmi Abd Ghani, Nazamid Saari

**Affiliations:** 1 Department of Food Science, Faculty of Food Science and Technology, Universiti Putra Malaysia, 43400 UPM Serdang Selangor, Malaysia; 2 Department of Food Technology, Faculty of Food Science and Technology, Universiti Putra Malaysia, 43400 UPM Serdang Selangor, Malaysia; 3 Section of Food Engineering Technology, Malaysian Institute of Chemical and Bio-Engineering Technology, Universiti Kuala Lumpur, Melaka, Malaysia

**Keywords:** Metabolomics, Monosaccharides, Polysaccharides, Applied microbiology, Biochemistry, Biotechnology

## Abstract

This study aimed to enhance natural gamma aminobutyric acid (GABA) production in yoghurt by the addition of simple sugars and commercial prebiotics without the need for pyridoxal 5′-phosphate (PLP) cofactor. The simple sugars induced more GABA production (42.83–58.56 mg/100 g) compared to the prebiotics (34.19–40.51 mg/100 g), with glucose promoting the most GABA production in yoghurt (58.56 mg/100 g) surpassing the control sample with added PLP (48.01 mg/100 g). The yoghurt prepared with glucose also had the highest probiotic count (9.31 log CFU/g). Simulated gastrointestinal digestion of this GABA-rich yoghurt showed a non-significant reduction in GABA content and probiotic viability, demonstrating the resistance towards a highly acidic environment (pH 1.2). Refrigerated storage up to 28 days improved GABA production (83.65 mg/100 g) compared to fresh GABA-rich yoghurt prepared on day 1. In conclusion, the addition of glucose successfully mitigates the over-use of glutamate and omits the use of PLP for increased production of GABA in yoghurt, offering an economical approach to produce a probiotic-rich dairy food with potential anti-hypertensive effects.

## Introduction

Yoghurt is a fermented form of milk with a thick consistency and has been consumed since ancient times. Nowadays, it is appreciated for its high nutritional value and positive health benefits, owing to the probiotic effects of the starter culture, i.e. lactic acid bacteria *Streptococcus thermophilus (S. thermophilus)* and *Lactobacillus delbrueckii* subsp*. bulgaricus (L. delbrueckii* subsp*. bulgaricus),* such as improved lactose digestion^1^, prevention of diarrhoea^2^ and stimulation of the gut immune system^3^. Globally, the yoghurt market was worth approximately 85.54 billion USD in 2019 and is forecasted to increase to 106.6 billion USD by 2024^4^.

Gamma aminobutyric acid (GABA) is a non-protein amino acid common in animals, plants, and microorganisms. In animals, it acts as the primary inhibitory neurotransmitter in the central nervous system, while it plays a key metabolic role in the Krebs cycle in plants and microorganisms^5^. Physiologically, GABA reduces stress^6^, inhibits cancer cell proliferation^7^, decreases blood pressure^8^ and prevents diabetes^9^. The biosynthesis of GABA occurs mainly through fermentation by microorganisms such as yeast, fungi, and bacteria. Typically, yoghurt starter cultures have poor glutamate decarboxylase (GAD) activity, for instance, *S. thermophilus* exhibited GAD activity in the range of 0.65 to 11 μmol/g/min of protein^10, 11^, while no GAD activity was reported for *L. delbrueckii* subsp*. bulgaricus* as reflected by extremely low GABA production^12^. Most lactic acid bacteria (LAB), namely *L. brevis, L. paracasei, L. plantarum,* and *Lactococcus lactis,* produce GABA through α-decarboxylation of glutamate via the enzymatic reaction of GAD, a pyridoxal 5′-phosphate (PLP) dependent enzyme^11^.

The need for a high concentration of glutamate (32–507 mM) as well as the presence of PLP (18–200 µM) are major obstacles for GABA production in food systems^10, 11^. Also, glutamate produces a salty/savoury taste at high concentrations that is unfavourable for yoghurt products while PLP cofactor is a costly ingredient. However, due to the various health benefits of GABA, yoghurt rich in GABA represents a value-added functional dairy product that can be conveniently consumed regularly. To the best of our knowledge, there are only a few studies regarding the effect of sugar on enhancing GABA production in culture medium^10, 11^ and no work has been reported on the effect of prebiotics in culture medium or these simple carbohydrates in an actual food system. Therefore, this study investigated (1) the effect of different carbohydrates (simple sugars and commercial prebiotics) on enhancing GABA production in yoghurt cultured using a mixture of two novel, self-cloned LAB strains (*L. plantarum* Taj-Apis362, previously engineered by Tajabadi et al.^17^ with a GAD activity of 167.2 μmol/ml/min, assigned as UPMC90 and UPMC91 by Institute of Bioscience, Universiti Putra Malaysia, Malaysia) to mitigate the over-use of glutamic acid and omit the need of PLP cofactor, (2) the stability of GABA-rich yoghurt during gastrointestinal digestion and 28-days of refrigerated storage, and (3) the metabolomics profile of the fermentation-derived biomolecules in yoghurt via ^1^H-nuclear magnetic resonance (NMR).

## Results and Discussion

### Effect of simple carbohydrates on GABA production in yoghurt

GABA production by microorganisms is affected by several factors including microbial genetic characteristics, culture conditions (temperature, pH, time) and media (presence of glutamate and PLP). Most studies reported the need for a high concentration of glutamate (32–507 mM) and the presence of PLP (18–200 µM) to achieve optimum GABA production in different food systems^10, 11^. To mitigate glutamate and PLP usage during fermentation, the current study improved the media by incorporating different simple carbohydrates, in the form of simple sugars and prebiotics, to maximise GABA production to determine the lowest effective concentration of glutamate (11.5 mM) for optimal GABA production. Previous studies had reported glutamate usage of 32 mM in fermented palm date residue^14^ and 80 mM in fermented milk^18^. This proves the efficient conversion of glutamate to GABA in yoghurt fermented by UPMC90 and UPMC91 LAB strains under pre-defined optimum conditions^19^.

**Figure 1:**
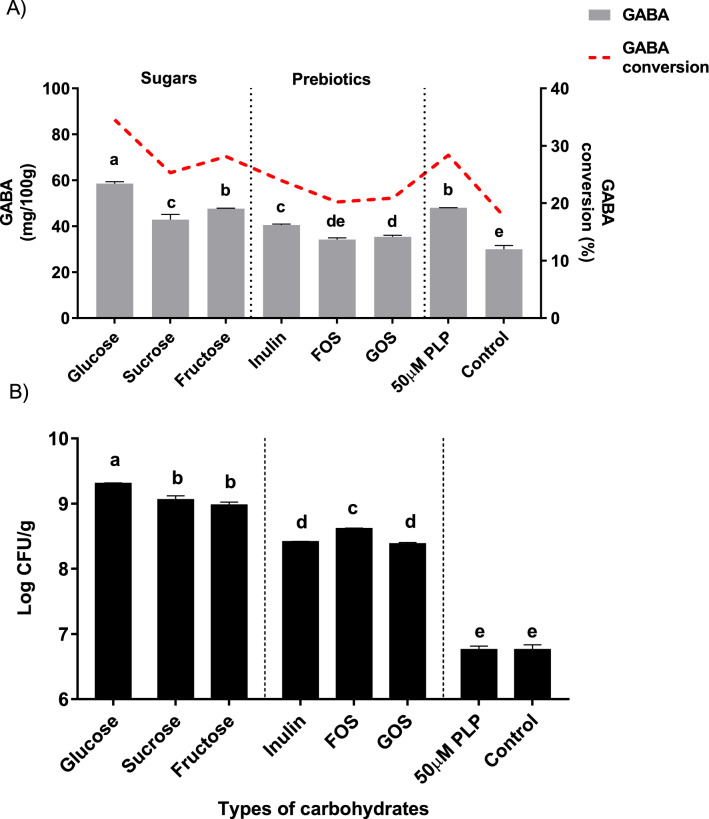
Effect of simple carbohydrates on (**A**) GABA content and conversion rate and (**B**) Viable cell count in yoghurt. Different letters indicate significant difference at *p* < 0.05.

The effect of different simple sugars and prebiotics on GABA content and conversion rate is depicted in Fig. 1A. Of the six simple carbohydrates, simple sugars induced more GABA production (42.83–58.56 mg/100 g) compared to prebiotics (34.19–40.51 mg/100 g). In particular, glucose significantly (p < 0.05) induced the highest GABA production in yoghurt (58.56 mg/100 g, conversion = 34.60%), favourably surpassing the control sample with added PLP (48.01 mg/100 g, conversion = 28.38%), a cofactor well known to promote GABA biosynthesis. In terms of viable cell count, glucose had the highest probiotic count (9.31 log CFU/g), followed by sucrose (9.06 log CFU/g) and fructose (8.98 log CFU/g) as depicted in Fig. 1B. The efficient utilisation of glucose by LAB strains (both UPMC90 and UPMC91) and starter culture to produce GABA and a high probiotic count was expected as glucose is readily phosphorylated to glucose-6-phosphate in the glycolytic cycle of the Embden-Meyerhof pathway and phosphoketolase pathway to achieve bacterial cell growth. In contrast, the other two simple sugars, namely sucrose and fructose, have to go through additional conversion steps in the phosphoenolpyruvate-dependent phosphotransferase system before conversion to pyruvate^20^, which then either splits into the GABA-shunt pathway to form GABA or continues to be decarboxylated through the glycolytic pathway to generate ATP, NADH and NADPH for cell growth^21^. The straightforward metabolism of glucose explains the rapid bacterial growth when the sugar is present, allowing growth to reach an exponential phase in a shorter time compared to other simple carbohydrates. The accumulation of active bacterial cells then contributes to increased secretion of GAD, thus higher enzymatic activity to convert glutamate into GABA. There was a positive correlation between viable cell count and GABA production during fermentation, i.e. a higher viable count is associated with more GABA formation.

Interestingly, the yoghurt containing prebiotics of inulin, fructooligosaccharides (FOS) and galactooligosacharides (GOS), had GABA contents and viable cell counts substantially lower than simple sugars, indicating poor utilisation of the prebiotics by the bacteria due to the high degree of polymerisation (DP) ranging from 2 to 65 in the prebiotics^22^. Similarly, Hernandez-Hernandez et al.^23^ reported reduced growth of *L. casei* ATCC11578 and *L. delbrueckii subsp. lactis* ATCC4797 strains in the present of GOS compared to glucose and lactulose (a synthetic disaccharide). Rayes^24^ also described that carbohydrates with high DP were poor substrates for bifidobacterial because prebiotics with a higher DP require cleavage into monosaccharides with a lower DP by extracellular bacterial enzymes before transportation into the cells for growth^25^.

Among the three tested prebiotics, inulin produced significantly more GABA than FOS and GOS but lower probiotic growth compared to FOS (Fig. 1A and 1B), suggesting that LAB strains of UPMC90 and UPMC91 with high GAD enzyme activity prefer to metabolise inulin over FOS. In accordance with our results, Choudhary et al.^26^ also showed that inulin was fermented at a higher rate than FOS by *L. paracasei* CD4 in soymilk. According to Sarbini and Rastall^27^, there are specific transport systems in LAB for trisaccharides and tetrasaccharides, indicating different metabolic capacity based on the type of substrate used. Also, each LAB strain has its preferred choice of prebiotics as substrates for fermentation depending on their respective genetic characteristics^28^. These findings explain the preferred utilisation of inulin over FOS by the UPMC90 and UPMC91 strains used in the current study, whereas the high viable cell count in FOS compared to inulin is mostly due to the abundance of starter culture, i.e. *S. thermophilus* and *L. delbrueckii ssp. bulgaricus*, in the sample.

PLP (50 µM, positive control) enhanced GABA production (48.01 mg/100 g, conversion = 28.38%) compared to yoghurt without any simple carbohydrates (negative control, 29.95 mg/100 g, conversion = 17.71%) and was higher than that reported by Yi and Chui^29^, who used 8.09 mM of PLP and 134 mM of monosodium glutamate in adzuki bean milk fermented by *L. rhamnosus* GG. The PLP sample also showed the lowest viable cell count (log 6.76 CFU/g) similar to the negative control, indicating no significant effect on bacterial cell growth compared to simple carbohydrates. This is in line with Li et al.^30^ who reported that PLP did not affect the growth of *L. brevis* NCL912. Therefore, the presence of PLP in yoghurt enhances GABA production but does not affect the growth of *L. plantarum* Taj-Apis362 strain UPMC90 and UPMC91. Among all tested simple carbohydrates, glucose displayed the highest substrate efficiency for bacterial metabolism, rapidly promoting cell growth which enhanced the conversion of glutamate into GABA during yoghurt fermentation, thus was selected for further studies as detailed below.

### Simulated digestion study on GABA-rich yoghurt

**Figure 2:**
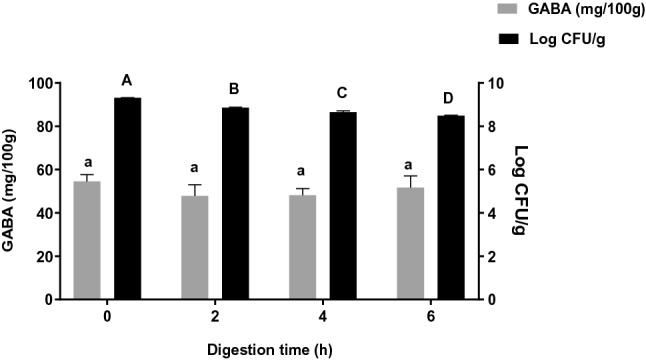
Effect of simulated digestion on GABA content and viable cell count in GABA-rich yoghurt. Different letters indicate significant difference (*p* < 0.05) for GABA content (small letters) and viable cell count (capital letters) during 6 h of simulated digestion.

In vitro gastrointestinal digestion is an effective and valid strategy to simulate digestion in the human gastrointestinal tract. Various mechanical, chemical and enzymatic actions occur within the human digestive tract to degrade food matrices, releasing nutrients that are readily absorbed by the body. In this study, the simulation was performed at pH 1.2 for the first 2 h to mimic stomach digestion, followed by increasing the pH to 6.8 for the next 4 h to mimic intestinal digestion. The results of GABA stability and the survival of probiotic cultures are shown in Fig. 2 as GABA content and viable cell count, respectively. No significant reduction of GABA was observed after 6 h of digestion. While gastrointestinal enzymes such as pancreatin and pepsin are available to perform hydrolysis in the simulated system, GABA is not digested because it is a non-protein amino acid, i.e. not a substrate for these enzymes. Instead, the structural integrity and stability of GABA are maintained through resistance to acidic pH. Similarly, a study by Le et al.^31^ revealed no significant reduction in GABA content in germinated soymilk after 2 h of simulated digestion at 37 °C.

In contrast, a slow reduction in probiotic viability was detected throughout hydrolysis, decreasing from 9.31 log CFU/g at 0 h to 8.49 log CFU/g at the end of 6 h digestion. A similar reduction was reported for *L. acidophilus* La-5 in fermented soy product^32^ and *B. animalis* subsp. *lactis* in goat milk ice cream^33^ under the same conditions. Probiotics are only deemed beneficial and useful when they tolerate the harsh acidic conditions in the stomach and withstand further digestion in the small intestine to reach the large intestine in viable form restoring gut microbial balance. While the viable count reduced significantly after digestion in the current study, it was maintained at 8.49 log CFU/g until the end of digestion, probably due to a complementary effect from the acid-resistance nature of bacteria and H^+^ ion-dependent GABA production. Coherently, Sanchart et al.^34^ reported the survival of LAB at pH < 2.5 for at least 2 h, while Wang et al.^35^ reported that GABA production involves the consumption of H^+^ ion from the extracellular environment, making it less acidic and favourable for probiotic survival. This study highlights the stability of UPMC90, UPMC91 and starter culture to maintain a viable cell count after simulated digestion.

## Storage stability study

### GABA content, viable cell count and pH

**Figure 3:**
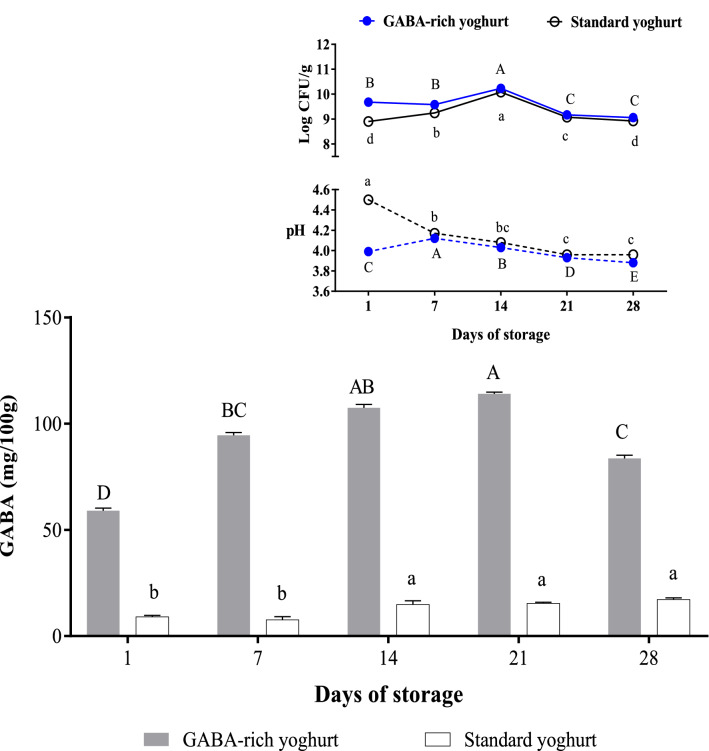
GABA content, viable cell count and pH of GABA-rich yoghurt compared to standard yoghurt during 28 days of storage at 4 °C. Different letters indicate significant difference (*p* < 0.05) among GABA-rich yoghurt (capital letters) and standard yoghurt (small letters) on different days of storage.

The GABA content and probiotic viability during storage are vital to ensure good bio-functionalities and health benefits for consumers without jeopardising the organoleptic properties (in terms of sourness measured as pH). The effects of 28-day refrigerated storage (2–4 °C) on GABA content, viable cell count and pH of GABA-rich yoghurt are illustrated in Fig. 3. A significant increase in GABA content was observed over 28-days storage compared to freshly fermented yoghurt on day 1 (59.00 mg/100 g), with the GABA content reaching a maximum of 113.95 mg/100 g on day 21, then decreasing to 83.65 mg/100 g on day 28. Despite this reduction, the GABA content was still higher than the initial value, indicating that refrigerated storage up to 28 days is acceptable but storage for 21 days is optimum. The increased GABA content over 28 days of storage reflects the continuous formation of GABA by the GABA-producing LAB strains of UPMC90 and UPMC91 as well as starter culture throughout the study period. The increasing GABA content during storage is in agreement with a previous finding^36^, whereby GABA increased from 5.71 mg/100 g (day 1) to 10.33 mg/100 g (day 14) during refrigerated storage of yoghurt cultured with starter culture and GABA-producing strains of *L. cremoris* and *L. lactis* O-114, *L. helveticus* Lh-B 02 and *L. rhamnosus* B-1445.

The viable cell count of GABA-rich yoghurt was initially recorded at 9.68 log CFU/g on day 1 and peaked at 10.23 log CFU/g on day 14, followed by a sharp reduction to 9.17 and further to 9.06 log CFU/g on day 21 and 28, respectively. While the viability reduced over time, it is still in accordance with the minimum standard count of 6.00 log CFU/g required for probiotic food recognition^37^, thus the GABA-rich yoghurt is a probiotic food for 28 days of storage. Interestingly, the highest GABA content was recorded on the reduced viable cell count on day 21 because GABA accumulates over time and is rarely converted into other products by microbes as GABA is not a preferred substrate. However, at the end of storage (day 28), the reduced GABA content may be attributable to *Saccharomyces cerevisiae*, a spoilage microbe known to utilise GABA^38^ and is often detected in yoghurt products^39, 40^. This strain contains enzymes that degrade GABA, i.e. GABA-permease, which is responsible for GABA uptake from the extracellular environment into the cell where it is converted to succinate by GABA-transaminase and semialdehyde dehydrogenase before entering the Krebs cycle for further assimilation as a carbon and/or nitrogen source^41, 42^. Since there were early signs of GABA reduction by day 28 of storage, extending the storage time longer than 28 days is not recommended. In terms of pH, a significant reduction was observed from pH 3.99 to 3.88 in GABA-rich yoghurt due to the production of lactic acid in the milk during refrigerated storage^43^. This pH range falls within the acceptable limits of pH 3.7–4.6 for commercial yoghurt, thus complying with the product specification requirement.

In standard yoghurt, GABA is minimally produced by the starter culture (a mixture of *S. thermophilus* and *L. delbrueckii ssp. bulgaricus*), recording values from 9.02 mg/100 g (day 1) to 17.16 mg/100 g (day 28) that are significantly lower than that of GABA-rich yoghurt. While the GABA-producing ability of these two strains has been acknowledged^44^, the conversion rate is low. Watanabe et al.^45^ reported poor GABA production (less than 5 mM) after 48 h of milk fermentation by the said starter culture. Therefore, the GABA-rich yoghurt had more GABA compared to standard yoghurt during 28 days of refrigerated storage.

## Water holding capacity and syneresis

**Figure 4:**
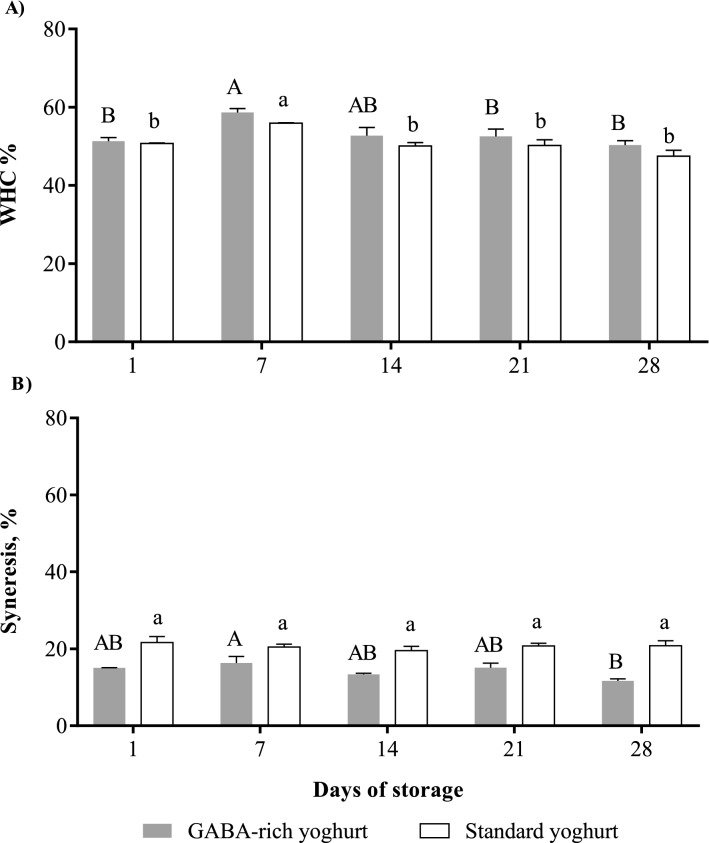
Effect of refrigerated storage on (**A**) WHC and (**B**) syneresis of GABA-rich yoghurt compared to standard yoghurt. Different letters indicate significant difference (*p* < 0.05) for GABA-rich yoghurt (capital letters) and standard yoghurt (small letters) on different days of storage.

Water holding capacity (WHC) and syneresis directly reflect the coagulum strength of yoghurt as a semi-solid, gel-like food product and are related to the textural and sensorial properties (mouthfeel, eating experience) of a product^46^. Syneresis, a common phenomenon in yoghurt, is considered unfavourable to consumers owing to the presence of exudate/fluid release from the food matrix. As shown in Fig. 4A, the WHC of GABA-rich and standard yoghurt recorded no significant changes over 28 days of storage, except for an increment on day 7, indicating the high stability of the yoghurt over time. The GABA-rich yoghurt exhibited a minor decrease in syneresis values (11.70–15.03%) compared to the standard yoghurt (20.79–21.63%) during storage (Fig. 4B). The low syneresis values are due to the acidic environment that enhances the gel network to resist syneresis during storage^47^. According to Lobato-Calleros et al.^48^ and Nguyen et al.^49^, the increasing WHC and reduced syneresis resulted from effective water molecule entrapment in the protein network. The addition of glucose did not affect the WHC and syneresis of the GABA-rich yoghurt and standard yoghurt.

## ^1^H-NMR Metabolomics analysis

Yoghurt comprises numerous biomolecules including proteins, lipids, sugars, amino acids, organic acids, fatty acids, minerals and volatile aroma compounds that contribute to the overall flavour and taste profile. In this study, both GABA-rich and standard yoghurt were freeze-dried before NMR spectroscopy to prevent signal interference from water molecules^50^. It is known that each microorganism induces metabolite changes via different metabolic pathways during fermentation^51^, therefore, a metabolomics approach based on ^1^H-NMR was used to compare the major metabolite profile of freeze-dried GABA-rich yoghurt (GY) and freeze-dried standard yoghurt (SY), both of which were fermented from milk, with SY containing only a starter culture of *S. thermophilus* and *L. delbrueckii ssp. Bulgaricus,* while GY contains additional GABA-producing LAB strains of UPMC 90 and UPMC91.

**Figure 5:**
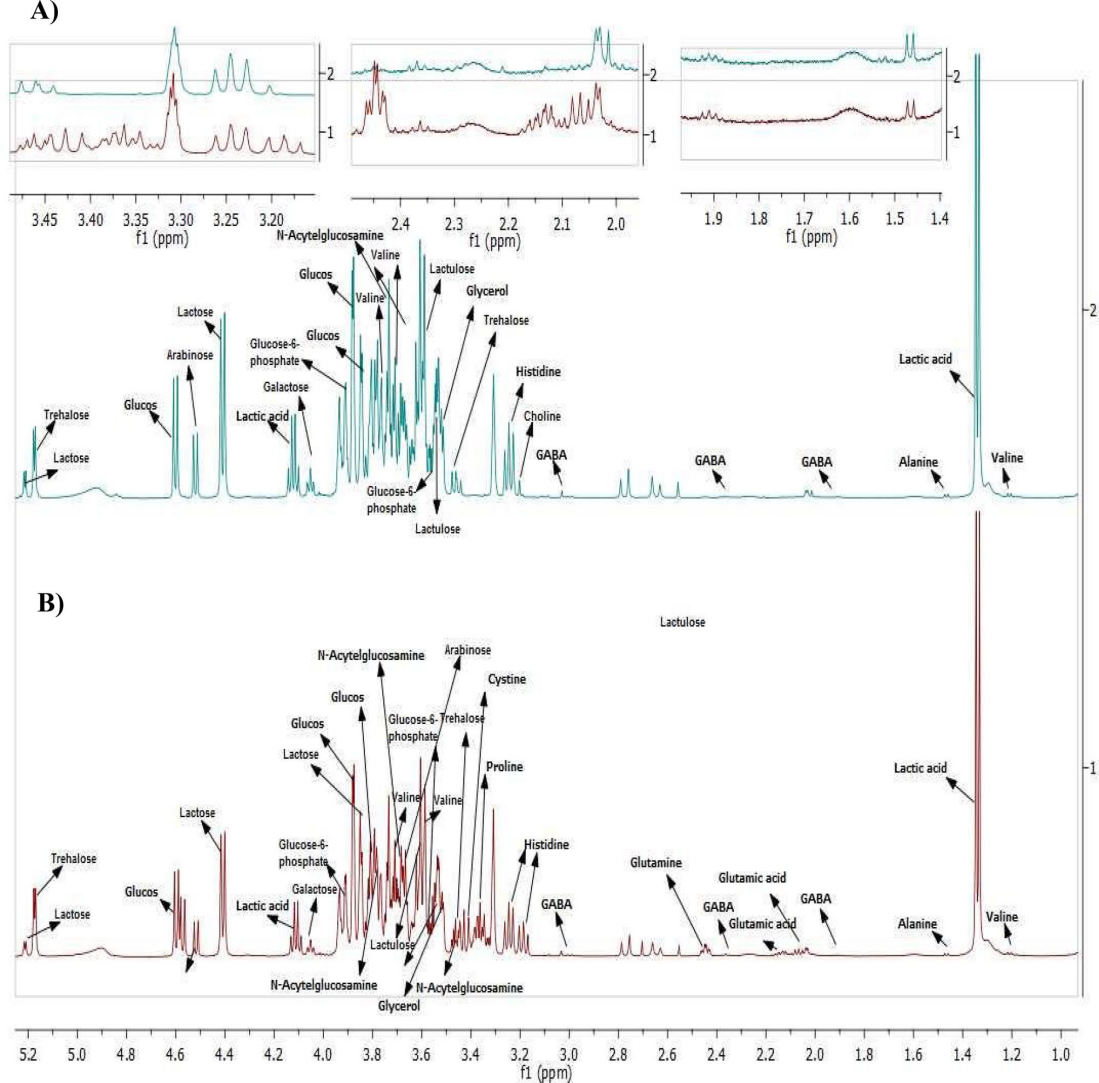
^1^H-NMR spectra of (**A**) freeze-dried standard yoghurt (SY) and (**B**) freeze-dried GABA-rich yoghurt (GY). The spectra was analysed using MestReNova software (Mestrelab, Santiago de Compostela, Spain). https://mestrelab.com/.

Figure 5 depicts the ^1^H-NMR spectra, showing that a total of 16 and 13 compounds were detected in GY and SY, respectively. The different metabolite profiles may be due to the strain-specific metabolic activities of GABA-producing LAB strains (UPMC90 and UPMC91). Similarly, a previous study reported that the free phenolic content varied during fermentation of whole-grain barley when different species of lactobacillus were used^52^. Table 1 tabulates the amino acid, sugar and organic acid content of GY and SY. Briefly, GY comprised seven amino acids including GABA, glutamine, alanine, histidine, proline, cysteine and valine, while SY comprised only four amino acids including GABA, alanine, histidine and choline. When the amino acid was present in both samples, GY showed a higher concentration than SY except for alanine. Similarly, GY demonstrated a higher GABA content (97.65 mg/100 g) than SY (25.10 mg/100 g), confirming that the addition of glucose increased natural GABA production by UPMC90 and UPMC91 LAB strains and that GABA was produced naturally by the starter culture in SY without the presence of GABA-producing strains.

**Table 1:** Metabolite profiles in freeze-dried GABA-rich yoghurt (GY) and freeze-dried standard yoghurt (SY) determined using ^1^H-NMR metabolomics-based analysis.

Metabolite	^1^H-NMR characteristic signals	GY	SY
		mg/100 g	
Glucose	δ 3.889 (dd), δ 3.824 (m), δ 3.889 (dd)	311.85	226.64
Glutamate	δ 2.04 (m), δ 2.119 (m), δ 2.341 (m), δ 3.748 (dd)	461.25	ND
GABA	δ 1.89 (m), δ 2.28 (t), δ 3.00 (t)	97.65	25.10
Glutamine	δ 2.13 (m), δ 2.44 (m),	290.53	ND
Alanine	δ 1.47 (d)	15.45	18.97
Histidine	δ 3.16 (dd), δ 3.23 (dd)	205.49	152.98
Choline	δ 3.189 (s)	ND	29.48
Proline	δ 3.33 (dt), δ 3.34 (m)	54.34	ND
Cysteine	δ 3.38 (dd)	177.02	ND
Valine	δ 3.61 (d), 3.7 (m),	38.80	14.41
Lactose	δ 3.55 (m), δ 3.79 (m), δ 4.44 (d), δ 5.22 (d)	346.41	1025.00
Lactulose	δ 3.582 (m), δ 3.732 (m)	189.54	307.23
Trehalose	δ 3.44 (t), δ 5.18 (d)	1174.00	842.39
Arabinose	δ 3.68 (m), δ 3.95 (m), δ 4.52 (d)	196.37	367.82
Galactose	δ 4.07 (t)	549.48	441.38
N-Acetylglucosamine	δ 3.47 (m), δ 3.65 (dd), δ 3.76 (m)	51.10	49.55
Lactic acid	δ 1.32 (d), δ 4.14 (d)	1338.00	2060.00

s = singlet; d = doublet; t = triplet; dd = doublet of doublets; m = multiplet; ND = not determined.

Seven sugars (glucose, lactose, lactulose, trehalose, arabinose, galactose and N-acetylglucosamine) were identified in both GY and SY samples, with more glucose (311.85 mg/100 g), trehalose (1174.00 mg/100 g) and galactose (549.48 mg/100 g) in GY compared to SY. The high concentration of glucose was due to the addition of this sugar into the fermentation medium as a GABA enhancer. Galactose is excreted into the medium when the microorganisms (starter culture and GABA-producing LAB strains) consume the glucose moiety of lactose in the milk^53^, leaving behind the galactose residue. The increased level of galactose in GY indicates the increased consumption of lactose as the preferred substrate in the starter culture co-inoculated with UPMC90 and UPMC91 LAB strains. Similar to our findings, a higher galactose content was observed when co-culturing *L. plantarum* WCFS1 with *S. thermophilus* and *L. delbrueckii ssp. bulgaricus* compared to that without *L. plantarum* WCFS1^54^. The lower amount of lactose correlates to the lower amount of lactic acid in GY, which can be explained by the heterofermentative metabolism of UPMC90 and UPMC91 strains favouring lactose utilisation for energy production while generating metabolites other than lactic acid, thus lowering the amount of lactic acid.

In contrast, the starter culture (*S. thermophilus* and *L. delbrueckii ssp. bulgaricus*) in SY produced more lactic acid due to homofermentative metabolism that generates lactic acid as the main end-product^55^. While yoghurt is widely recognised as suitable for lactose intolerant individuals, the significantly lower amount of lactose in GY (346.41 mg/100 g) compared to SY (1025.00 mg/100 g) provides an additional benefit to patients suffering from severe lactose intolerant symptoms. In short, the metabolites were produced due to major structural alteration of milk components through two biochemical pathways, (i) glycolysis whereby carbohydrate is converted into lactic acid or other metabolites, and (ii) proteolysis whereby casein is hydrolysed into a peptide or free amino acid^56^.

## Conclusion

The current study is the first to report the effect of simple sugars and prebiotics in enhancing natural GABA production in a food system (yoghurt). The addition of glucose (2% w/v) enhanced GABA production in a very low concentration of glutamate (11.5 mM) without the need to add a PLP cofactor. The simulated gastrointestinal and storage studies revealed the good stability of GABA and viable cell count under gastrointestinal conditions as well as refrigerated storage up to 28 days, meeting the minimum requirement of 6.00 log CFU/g for recognition as a probiotic food. The addition of glucose had no adverse impact on the water holding capacity compared to standard yoghurt. This study successfully mitigates the over-use of glutamate and omits the use of the expensive PLP cofactor in the production of GABA-rich yoghurt, offering an economical approach to produce a probiotic-rich, functional dairy food with prospective stress management and cardiovascular disease prevention properties. Before human consumption, a rigorous risk assessment involving the identification of potentially hazardous substances and the likelihood of the occurrence of adverse effects from genetic engineered strains should be conducted on the GABA-rich yoghurt to ensure product safety.

## Materials and methods

### Materials

Non-fat skimmed milk powder (Sunlac brand) and pasteurised fresh milk (Goodday brand) were purchased locally. Food grade commercial prebiotics (90–95% purity), inulin and galactooligosaccharides (GOS) were purchased from CK Chemical Sdn Bhd and fructooligosaccharides (FOS) were purchased from Greenfinite Sdn Bhd. MRS agar and MRS broth were obtained from HiMedia Laboratories Pvt. Ltd. (Mumbai, India). Glutamate, GABA standard and triethylamine were obtained from Merck KGaA (Darmstadt, Germany). Methanol-d_4_, deuterium oxide (D_2_O) and sodium deuteroxide (NaOD) for NMR analysis were purchased from Cambridge Isotope Laboratories, Inc. (Tewksbury, MA, USA). All other chemicals were analytical or HPLC grade.

### Preparation of starter culture and GABA-producing LAB strains

Two *L. plantarum* Taj-Apis362 strains possessing high intracellular GAD activity (UPMC90) and high extracellular GAD activity (UPMC91) were obtained from the culture collection of the Institute of Bioscience, Universiti Putra Malaysia and routinely stored in sterile MRS broth at -80 °C as a stock culture. These strains were characterised previously, whereby the wild-type *L. plantarum* Taj-Apis362 was isolated from the stomach of the honeybee *Apis dorsata* and used as a host for GAD gene overexpression to produce UPMC90 and UPMC91 strains^17^. All procedures involving the use of *L. plantarum* Taj-Apis strains were approved by the National Board of Biosafety, Ministry of Natural Resources and Environment, Malaysia (approval no. JBK [S]-602–1/2/207). Commercial starter culture (Lactina brand) containing *S. thermophilus* and *L. delbrueckii ssp. bulgaricus* was obtained from YoghurtBio (Sofia, Bulgaria). Reconstituted skim milk was prepared by mixing commercial pasteurised fresh milk and skimmed milk powder to reach 16% non-fat dry matter, then heat-treated at 80–85 °C for 30 min^49^, cooled to 4 °C on ice before aliquoting into 250-mL screw-capped Schott bottles and storage at 4 °C for 24 h before use. At the beginning of each fermentation cycle, i.e. production of a new batch of yoghurt sample, starter culture and LAB strains were prepared fresh from the stock. The starter culture was inoculated into sterilised reconstituted skimmed milk and incubated at 42 °C for about 6 h until the pH reached 4.5–4.6. LAB strains were streaked for single colony isolation on MRS agar, then transferred to 10 mL MRS broth, incubated for 18 h at 37 °C to allow cell growth, and transferred to sterilised reconstituted skimmed milk for sub-culture for 22–24 h. At the end of incubation, the coagulated milk was employed as inoculum for yoghurt production.

### Addition of sugars and prebiotics to yoghurt

Eight samples of yoghurt were prepared: negative control (yoghurt with glutamate only), positive control (yoghurt with glutamate + 50 µM of PLP cofactor), and yoghurt with glutamate + 2% (w/v) of glucose, sucrose, fructose, inulin, FOS and GOS, respectively. The amount of sugar/prebiotic (2%) was selected based on previous studies to induce GABA in various samples of kimchi^57^, cornhub hydrolysate^58^ and skimmed milk^59^. Briefly, the yoghurt samples were prepared by co-inoculating the starter culture and GABA-producing LAB strains (viable count of 10^6^ CFU/g) simultaneously at a ratio of 2:1 w/w into fresh sterilised reconstituted skimmed milk, then 11.5 mM glutamate was added and fermentation was allowed for 7.25 h at 39°C^19^. Upon completion, samples were rapidly cooled in an ice bath to stop fermentation and stored at 2–4 °C until further analysis.

### Determination of GABA content

GABA and glutamate were determined following the method previously described by Tajabadi^17^ via HPLC (Shimadzu LC 20AT, Shimadzu Corporation, Kyoto, Japan) equipped with an oven (model CT0-10ASVP), pump system and PDA detector (model SPD-M20A). A Chromolith RP-18 endcapped separation column (100 mm length × 4.6 mm internal diameter, Merck KGaA, Darmstadt, Germany) was used for this analysis. The yoghurt sample was centrifuged at 10,000 × g for 15 min at 4 °C and 10 μL of the supernatant was placed into a small Durham tube and evaporated under vacuum for 40 min. Then, the dried supernatant was dissolved in 20 μL of a mixture of ethanol/water/triethylamine solution at a ratio of 2:2:1 and vacuum evaporated for another 40 min, followed by the addition of 30 μL of a mixture of ethanol/water/triethylamine/phenylisothiocyanate solution at a ratio of 7:1:1:1 and left for 20 min at room temperature to allow phenylisothiocyanate-GABA formation. The sample was vacuum evaporated for 40 min to remove excess reactant.

The derivatised sample was then diluted and subjected to HPLC analysis. The mobile phase A was prepared by dissolving 8.205 g of sodium acetate, 0.5 mL of trimethylamine and 0.7 mL of acetic acid in 1000 mL of deionised water, then the pH was adjusted to 5.8 using 0.1 M sodium hydroxide. Meanwhile, mobile phase B was prepared by mixing acetonitrile with deionised water at a ratio of 60:40 (v/v). Both mobile phases were filtered through a 0.45 μm membrane filter. The sample (5 µL) was injected and eluted at a flow rate of 0.6 mL/min using isocratic elution of 80% mobile phase A + 20% mobile phase B. Compound detection was performed using a diode array detector at λ = 254 nm. The GABA and glutamate contents were calculated by comparing the sample peak area with the GABA standard and glutamate standard, respectively.

### Viable cell count

Bacterial enumeration was performed using the pour plate method. Firstly, 1.0 g of yoghurt sample was diluted with 9.0 mL of sterile peptone water. Subsequently, a tenfold dilution was made using peptone water, and 0.1 mL of the diluted sample was spread on MRS agar and cultured at 37 °C for 48 h to allow cell growth. The colonies appearing on the plates were then counted, multiplied by the dilution factor, and expressed as log colony-forming unit per g (log CFU/g).

### Gastrointestinal stability study (simulated digestion)

From the six yoghurt samples with different sugars and probiotics, the sample with the highest GABA content and viable cell count was selected for further product performance evaluation and characterisation as follows: gastrointestinal stability study, 28-days storage stability study and metabolomics profiling. Simulated digestion was performed following the method described by Auwal^60^. Two solutions were prepared, simulated-gastric-fluid (SGF) and simulated-intestinal-fluid (SIF). The SGF was prepared by mixing 20 mg/mL of pepsin, 350 µL of concentrated HCl and 0.1 g of NaCl in deionised water to a total volume of 50 mL and the pH was adjusted to 1.2. Next, 1 mL of the SGF solution was added to 3 mL of yoghurt and incubated at 37 °C in a water bath shaker for 2 h. Meanwhile, the SIF solution was prepared by mixing 34 mg/mL of KH_2_PO_4_, 3.85 mL of NaOH (200 mM) and 0.5 g of pancreatin in deionised water to a final volume of 50 mL and the pH was adjusted to 6.8. Then, 1 mL of the SIF solution was added to the reaction mixture and re-incubated for 4 h under the same conditions. Aliquots (1 mL) were taken at 0, 2, 4 and 6 h and boiled at 100 °C for 10 min to inactivate enzymes and stored at − 20 °C for GABA content. For viable cell count, aliquots of 1 mL were also taken at 0, 2, 4 and 6 h and rapidly cooled before storing at -20 °C.

### pH determination

The pH value of yoghurt samples was measured using a pH meter (model S20 SevenEasy, Mettler-Toledo GmbH, Columbus, OH, USA).

### Water holding capacity and syneresis

The WHC of yoghurt was determined according to the modified procedure described by Abdelmoneim^61^. Yoghurt sample of 10 g (W_1_) was centrifuged at 5000 g for 10 min at 4 °C, the supernatant was collected and weighed (W_2_). The WHC (%) was calculated as follows:

1 $${\text{WHC }} = \frac{{W_{1} - W_{2} }}{{ W_{1} }} \times 100\%$$

Syneresis was determined according to the method of Aguilera^62^. Briefly, 10 g of yoghurt sample (W_1_) was centrifuged at 700 g for 10 min at 4 °C, the supernatant was collected and weighed (W_2_), and the degree of syneresis (%) was calculated as follows:

2 $${\text{Degree}}\;{\text{of}}\;{\text{syneresis }} = \frac{{W_{2} }}{{ W_{1} }} \times 100\%$$

### Metabolomics profiling (^1^H-NMR analysis)

Yoghurt samples were freeze-dried and subjected to ^1^H-NMR analysis as described by Muhialdin^63^. A total of 10 mg of freeze-dried yoghurt was mixed with 0.375 mL of CH_3_OH-d_4_ and 0.375 mL of KH_2_PO_4_ buffer in D_2_O containing 0.1% trimethylsilyl propionate as an internal standard. The pH was adjusted to 6 with NaOD. The mixture was vortexed for 1 min, sonicated in an ultra-sonicator at 30 °C for 15 min and centrifuged at 13,000 rpm for 10 min. Aliquots of supernatant (600 μL) were transferred to an NMR tube for ^1^H-NMR analysis. Spectra were recorded at 26 °C on a spectrometer (model UNITY INOVA 500, Agilent Technologies Inc., Santa Clara, CA, USA) using a frequency of 500 MHz and tetramethylsilane was used as an internal standard. The spectra were automatically phased and bucketed with standard bins of δ 0.05 ranging from region δ 0.50 to 10.00. The metabolites were identified using Chenomx software version 8.5 (Chenomx Inc., Edmonton, Canada). The residual methanol region (δ 3.28 to 3.33) and water region (δ 4.70 to 4.96) were excluded from the analysis. Two-dimensional ^1^H–^1^H J-resolved and Heteronuclear Multiple-Bond Correlation (HMBC) were employed for metabolite identification. Six replicates were examined for each yoghurt sample.

### Statistical analysis

Analysis of variance (ANOVA) followed by Duncan’s test and 2-sample t-test were used to evaluate means at significant difference of p < 0.05 using Minitab software version 16 (Minitab Inc., State College, PA, USA). All values were reported as means ± standard deviation from at least triplicate determinations.
